# Adverse pregnancy and birth outcomes in sexual minority women from the National Survey of Family Growth

**DOI:** 10.1186/s12884-022-05271-0

**Published:** 2022-12-09

**Authors:** Veronica Barcelona, Virginia Jenkins, Laura E. Britton, Bethany G. Everett

**Affiliations:** 1 Columbia University School of Nursing, 560 W 168thStreet, Mail Code 6, New York, NY USA; 2 The University of Utah, Salt Lake City, UT USA

**Keywords:** Preterm birth, Low birthweight, Sexual and gender minorities, Hispanics, African Americans

## Abstract

**Background:**

Few studies have examined how multiple marginalized identities are associated with adverse pregnancy and birth outcomes, especially for Black and Hispanic sexual minority women. Sexual minorities are people who identify as lesbian, gay, bisexual or transgender (LGBT). The purpose of this study was to examine differences in adverse pregnancy (i.e., miscarriage) and birth outcomes (i.e., preterm birth, low birthweight, and stillbirth) in a national sample of women by race and ethnicity, and sexual minority status (LGBT identification and same-sex sexual behavior).

**Methods:**

We conducted a cross-sectional analysis of the National Survey of Family Growth (NSFG). The unit of analysis was pregnancy, not participants. In this study, we examined pregnancies to participants who identified as heterosexual, lesbian, and bisexual, by race and Hispanic ethnicity. We also studied sexual behaviors to categorize participants as women who have sex with women (WSW) and women who have sex with men (WSM). Outcomes included preterm birth, low birthweight, miscarriage, and stillbirth. We employed logistic and linear regression analyses for analyses using STATA.

**Results:**

We studied 53,751 pregnancies, and 9% of these occurred in people who identified as heterosexual, but had engaged in sexual activity with a female partner (heterosexual-WSW), 7% in those identifying as bisexual, and 1% to women who identified as lesbian. Pregnancies ended in preterm birth (10.7%) and low birthweight (9.0%), stillbirths (2–4%), and miscarriages (17–21%) in sexual minority women. We observed that pregnancies reported by Hispanic lesbian women had a higher birthweight (β = 10.71, SE = 4.1, *p*-value = 0.01) compared to infants born to Hispanic heterosexual-WSM. Pregnancies to lesbian women were significantly more likely to end in stillbirth (aRR = 3.58, 95% CI 1.30,9.79) compared to heterosexual-WSM. No significant differences were noted in risk of adverse birth outcomes by sexual orientation for NH Black or Hispanic women.

**Conclusion:**

In this sample, preterm births were less likely to occur among heterosexual-WSW than in heterosexual-WSM. Pregnancies to lesbians and bisexual women were more likely to end in miscarriage or stillbirth than heterosexual WSM. Lesbian Hispanic women reported higher birthweights compared to heterosexual-WSM Hispanic women. More research should be done to further understand these findings.

## Background

Inequities in adverse pregnancy and birth outcomes in the United States (U.S.) have been well-documented for decades among women (and pregnant people). Despite knowledge of social, medical, and obstetric risk factors, non-Hispanic (NH) Black women continue to experience 2 to 3 times the rates of preterm birth, low birthweight, and infant mortality than NH White women [1–4]. From 2019–2020, the percentage of preterm births (< 37 weeks gestation) significantly declined for NH White women (9.26% to 9.10%) and Hispanic women (9.97% to 9.84%), but not for NH Black women (14.39% to 14.36%) [2]. Pregnancy loss is less studied, yet available data demonstrate similar patterns of inequity by race and ethnicity. The perinatal mortality rate is defined as late fetal death at ≥ 28 weeks or and early neonatal death < 7 days [5]. Rates of perinatal mortality were significantly higher among NH Black women (10.66 per 1000 live births and fetal deaths at 28 weeks of gestation or more) and Hispanic women (5.35 per 1000) than NH White women (4.98 per 1000).[5] Globally, 15.3% of all recognized pregnancies end in miscarriage, with a higher likelihood reported among women who identify as NH Black [6]. There is no national surveillance for miscarriage in the U.S., and it is particularly challenging to measure because many miscarriages may go unrecognized, without the pregnant person realizing they conceived. However, evidence from a community cohort suggests that racial and ethnic disparities in miscarriage exist [7].

People who are sexual minorities face unique barriers to accessing perinatal health care as well. According to a recent Gallup poll, 5.6% of the U.S. population identifies as lesbian, gay, bisexual, or transgender (LGBT) [8]. An estimated 19% of sexual minority women (lesbian, gay, or bisexual; SMW) are raising children, and among female same-sex couples, 71.9% are raising a genetically related (biological) child [9]. Descriptive evidence suggests that inequities exist in SMW compared to heterosexual women’s adverse pregnancy outcomes. An analysis of a population-based sample found that in comparison to heterosexual women who have sex with men, lesbian women had a higher odds of miscarriage, stillbirth, low birth weight, and very preterm birth [10].  Bisexual women also had higher rates of miscarriage and very low birth weight than heterosexual women in that sample [10]. In contrast, an analysis of birth certificate data from Massachusetts revealed no differences in fetal growth nor in preterm birth among women in same-sex marriages compared to heterosexual marriages [11]. New data from California, however, found that women in relationships with women, as defined by having to mothers on birth certificate records, showed that these births were more likely to have multiple adverse outcomes and an increased risk of maternal morbidities to the birthing mothers compared to births where both a mother and father were identified on the birth certificate record [12]. Differences exist, however, in methodology and definitions of sexual minority status, limiting comparability between studies.

SMW’s health in pregnancy may be affected by minority stress, medical mistrust, or difficulties accessing quality care, all of which emerge from the long history of anti-LGBT discrimination, marginalization, and persecution [13, 14]. Sexual minority adults report avoiding health care because they anticipate discrimination (18%) and actually experiencing discrimination in healthcare encounters (16%) [15]. In addition, the likelihood of adverse pregnancy outcomes may be greater for SMW because they are more likely than their heterosexual counterparts to report negative preconception health indicators, such as binge drinking, substance use, diagnosis of a sexually transmitted infection, or depression [16, 17]. Furthermore, SMW may face socioeconomic barriers to accessing health care and achieving optimal health behaviors while pregnant. Compared to their heterosexual peers, SMW are more frequently: unemployed (9% vs 5%), uninsured (15% vs 12%), and low-income (< $24,000 annual income) (25% vs 18%) [18]. Less frequently studied are the psychological strengths of the LGBT community, including strong social networks, community and organizational ties, and flexibility in the conception of one’s self and identity [19].

There is a growing body of research further examining perinatal inequities among multiply minoritized people by race, ethnicity, sexual orientation, and gender identity. Pregnant people who identify as SMW and NH Black or Hispanic may experience heightened stress, maladaptive stress coping responses (such as smoking), and medical mistrust that reduces engagement in care [20, 21]. Kimberlé Crenshaw’s theory of intersectionality asserts that people experience injustices uniquely based on how these multiple social constructs overlap within systems and structural inequities [22]. Previous studies have applied an intersectional approach to examining multiple marginalized identities, examining masculine and feminine presentation, race, ethnicity, and experiences of discrimination, victimization and stigma [10, 23]. A recent analysis of the National Longitudinal Study of Adolescent to Adult Health guided by the intersectionality model found that sexual minority women exhibited lower rates of preterm birth overall, but the risk varied by race [24]. NH Black sexual minority women had 11.74 times the risk of preterm birth and Hispanic sexual minority women had 6.52 times the risk of preterm birth compared to NH White heterosexual women. Similar trends were observed for low birth weight [24]. As there are few large, nationally representative datasets on pregnancy and birth outcomes that simultaneously measure sexual and gender minority status, there is a need for additional studies to replicate these findings.

Therefore, in order to better understand how multiple marginalized identities are associated with adverse pregnancy and birth outcomes, we examined differences in adverse pregnancy (i.e., miscarriage) and birth outcomes (i.e., preterm birth, low birthweight, and stillbirth) in a national sample of women by race and ethnicity, and sexual minority status. We hypothesized that pregnant people with multiple marginalized identities would have worse pregnancy and birth outcomes than their counterparts who represented White culture. As the dataset used for this analysis did not comprise the full extent of intersectionality (i.e. institutions, systems, structures), we focused on individual-level identities as indicators of social position within the context of social inequities and systems of oppression.

## Methods

We conducted a cross-sectional study of data from the National Survey of Family Growth (NSFG). The NSFG is an annual survey that began in 1973, which collects a combination of self-administered surveys and in-person interviews. This nationally representative sample includes civilian, non-institutionalized reproductive-aged U.S. women between the ages of 16 and 45 (Centers for Disease Control and Prevention, 2018). In 2006, the NSFG began to include questions measuring sexual identity and same-sex sexual behavior. The NSFG has a response rate of 69% for recent data releases, and includes detailed reproductive histories for all reported pregnancies and their outcomes. Survey data was collected using Computer-Assisted Personal Interviews (CAPI) methods, administered by interviewers but completed on their own. All information provided in the surveys was self-reported [25]. Institutional review board approval was not obtained for this secondary analysis.

We restricted our analytic sample to pregnancies reported by NSFG participants between 2006 and 2017. Our total eligible sample was 53,751 pregnancies. For analyses restricted to live births, our total eligible sample was 36,374. We limited our analysis to singleton pregnancies of participants who were 1) currently not pregnant, and 2) who identified their race/ethnicity as NH Black, NH White, and Hispanic as distinct groups, as there were insufficient sample sizes for participants in the “other” category (*n* = 3,072, 5%, and describe which groups were represented in “other”, i.e. Asian, Native American, etc.).

The unit of analysis was pregnancy, and we applied the NSFG’s complex sampling frame, clustering by mother to account for multiple births reported to a single woman. All pregnancy history and birth outcomes were self-reported and collected retrospectively at the same time as demographic information by via CAPI.

### Exposures

*Sexual Orientation* was our primary predictor variable and was measured categorically, combining both sexual behavior and orientation measures as gender identity data were not available. Respondents were asked, "Do you think of yourself as heterosexual or straight; homosexual, gay or lesbian; or bisexual?" Respondents were also asked, "Have you ever had any sexual experience of any kind with another female?" From these questions, we created a four-category variable including heterosexual-identified with only male sexual partners (heterosexual-women who have sex with men (WSM)], heterosexual-identified with female partners (heterosexual-WSW), bisexual, and lesbian.

*Race/ethnicity* was constructed as a three-category variable, derived from two survey items that first asked respondents, “Are you Hispanic or Latina, or of Spanish origin?” followed by a survey item that asked “What is your race?” Respondents were recoded into mutually exclusive categories, NH White, NH Black, and Hispanic.

*Maternal Age* was derived from the participant's age at the time of the interview and the year the pregnancy ended.

*Education* was measured categorically, including less than a high school diploma, completed high school, some college, or a college degree.

Socioeconomic status was operationalized as *Percent of Federal Poverty Line (FPL), which* was measured as a categorical variable that captured whether household income adjusted for household size was < 100% of the FPL, [greater than or equal to] 100% and < 200% FPL, or [greater than or equal to] 200% FPL.

*Nativity* was measured as a dichotomous variable, with respondents who were born in the U.S. coded as 0 and those born outside the U.S. coded as 1.

*Previous Preterm Birth* was a variable created using the roster and birth information to capture whether a participant had previously reported a preterm birth prior to the index pregnancy.

### Outcomes

*Preterm birth* was derived from a survey item that asked respondents “A preterm delivery is one that occurs at 36 weeks or earlier in pregnancy. As far as you know, did you have a preterm delivery?”.

*Low birthweight* was a NSFG recode derived from the item, “Did (she/he) weigh 5.5 pounds or more, or less than 5.5 pounds.” If the respondent stated that their infant was less than 5.5 pounds at birth, they were coded as being low birthweight. If the respondent stated that their infant was more than 5.5 pounds at birth, they were coded as not being low birthweight.

*Birthweight* was measured using survey items that asked respondents to self-report an infant’s birthweight in pounds and ounces. We then recoded this to represent birthweight in ounces alone.

*Miscarriage* was captured using the item, “In which of the ways shown on Card 13 did the pregnancy end?” Answer choices included miscarriage, stillbirth, abortion, ectopic or tubal pregnancy, live birth by c-section, and live birth by vaginal delivery. If the respondent reported that the pregnancy ended in a miscarriage, that pregnancy was dichotomously coded as a miscarriage.

*Stillbirth* was measured using the same question and responses as miscarriage. If the respondent reported that the pregnancy ended in a stillbirth or that the pregnancy ended after 20 weeks of gestation, that pregnancy was dichotomously coded as a stillbirth.

We controlled for a variety of covariates associated with our exposure and outcome measures. These included socioeconomic status, education, nativity, and age. To examine how multiple stigmatized identities may impact findings, we also stratified by race.

### Statistical analysis

We first conducted bivariate analyses of preterm birth by sexual orientation. Next, we employed logistic regression models for the dichotomous outcome measures including preterm birth, low birthweight, miscarriage, and stillbirth. Linear regression models were used for continuous birthweight. In unadjusted analyses, we examined the association between sexual orientation [heterosexual- WSM; heterosexual-WSW), bisexual, or lesbian] and each birth outcome [preterm birth, low birthweight, miscarriage, and stillbirth]. We then conducted adjusted analyses controlling for maternal age, education, FPL, nativity, and previous preterm birth. We also include results stratified by race and ethnicity. All models were estimated using STATA Standard Edition version 14.2 and adjusted for NSFG population weights. Our unit of analysis was the pregnancy; therefore, we clustered on participants to account for the non-independence of pregnancies reported by each individual.

## Results

In Table 1, we present results for bivariate analyses for the total sample by sexual orientation (*N* = 53,751). Nine percent (n = 5,020) of pregnancies occurred in people who identified as heterosexual, but had in engaged in sexual activity with a female partner (heterosexual-WSW), 7% (n = 3,592) were to participants who identified as bisexual, and 1% (*n* = 486) were to women who identified as lesbian. Approximately 10% of births to heterosexual-WSM were preterm, while 8.3% were preterm for births to lesbian-identified women. Nearly 11% of births were low birthweight for heterosexual-WSM, compared to 11.2% for heterosexual-WSW and bisexual women, and 12.0% for lesbian women. Higher percentages of pregnancies ending in stillbirth were reported by SMW than heterosexual-WSM; 1.97% of pregnancies reported by heterosexual-WSM women ended in stillbirth compared to 2.5% 3.6% and 4.4% of pregnancies reported by heterosexual-WSW, bisexual, and lesbian women, respectively. Pregnancies also ended in miscarriage at a higher frequency for SMW, with rates of 17.4% heterosexual-WSM, 21.4% for bisexual women, and 18.4% for lesbian women compared to 14.97% for heterosexual-WSM.

**Table 1:** Bivariate characteristics of participants by sexual orientation, National Survey of Family Growth (NSFG), 2006–2019 (*N* = 53,751)

	Total Pregnancies:		Heterosexual- WSM		Heterosexual-WSW		Bisexual		Lesbian		Missing	
	53,751/36,374 Live Births		Pregnancies: 44,653		Pregnancies: 5,020		Pregnancies: 3,592		Pregnancies: 486			
	n	%	n	%	N	%	n	%	n	%	n	%
Total pregnancies (%)												
Preterm birth												
Yes	4168	10.77	3529	10.71	346	10.85	263	11.84	30	8.38	0	0
No	34,538	89.23	29,409	89.29	2843	89.15	1958	88.16	328	91.62		
Low birthweight												
Yes	3301	9.08	2764	9.67	291	11.28	215	10.96	31	12.02	0	0
No	33,073	90.92	28,437	91.14	2,718	90.33	1,691	88.72	227	87.98		
Birthweight, ounces [mean, s.e.]	115.66	0.13	115.81	0.14	115.1	0.44	113.6	0.62	117	1.74	6,762	18.59
Stillbirth (> 20 weeks)												
Yes	787	2.12	626	1.97	77	2.50	72	3.64	12	4.44	16,590	30.86
No	36,374	97.88	31,201	98.03	3,009	8.27	1,906	96.36	258	95.56		
Miscarriage (< 20 weeks)												
Yes	7,929	15.65	6,327	14.97	833	17.44	697	21.48	72	18.41	3,084	5.74
No	42,738	84.35	35,928	85.03	3,943	82.56	2,548	78.52	319	81.59		
Maternal age [mean, s.e.]	24.25	0.03	24.48	0.03	23.66	0.08	22.3	0.09	22.52	0.29	1,834	
Race												
Non-Hispanic White	23,948	44.55	19,015	42.58	2,848	56.73	1,901	52.92	184	37.86	0	0.00
Non-Hispanic Black	14,271	26.55	11,770	26.36	1,268	25.26	1,026	28.56	207	42.59		
Hispanic	15.532	28.90	13,868	31.06	904	18.01	665	18.51	95	19.55		
Education												
< High school/High school graduate	24,570	45.71	20,803	46.59	1,683	33.53	1,828	50.89	256	52.76	0	0.00
Some college	14,701	27.35	11,513	25.78	1,888	37.61	1,152	32.07	148	30.45		
College graduate	14,480	26.94	12,337	27.63	1,449	28.86	612	17.04	82	16.87		
Percent of the federal poverty line												
0–100%	19.859	36.95	16,394	36.71	1,719	34.24	1,574	43.07	199	40.95	0	0.00
100–200%	13,737	25.56	11,365	25.45	1,311	26.12	944	26.28	117	24.07		
> 200–300%	20,155	37.50	16,894	37.83	1,990	39.64	1,101	30.65	170	34.98		
Foreign born												
Yes	9,390	17.47	8,895	19.92	239	4.76	188	5.23	68	13.99	0	0.00
No	44,361	82.53	35,758	80.08	4,781	95.24	3,404	94.77	418	86.01		
Previous preterm birth												
Yes	3,137	5.84	2,724	6.10	235	4.68	160	4.45	18	3.70	0	0.00
No	50,614	94.16	41,929	93.90	4,785	95.32	3,432	95.55	468	96.30		

We also found some differences in demographics by race and ethnicity. The mean maternal age was 24.2 years, and 42.5% (*n* = 207) of lesbians identified as NH Black, compared to 26.3% of heterosexual-WSW. About 27% of heterosexual-WSM reported a college degree compared to only 17.0% of bisexual and 16.8% of lesbian women, respectively. Groups also differed on nativity status with 17.4% of heterosexual-WSM reporting they were foreign born, compared to only 4.7% of heterosexual-WSW and 5.2% of bisexual women. Pregnancies reported by heterosexual-WSW were more frequently occurring in women who had a previous preterm birth (6.1%) compared to other groups; this may be indicative of higher parity among this population.

Linear regression analyses (Table 2) showed no significant differences in birthweight by sexual orientation, race or ethnicity in both unadjusted and adjusted models. We did observe, however, that compared to infants born to Hispanic heterosexual-WSM, pregnancies reported by Hispanic lesbian women resulted in higher birthweight after adjustment for confounders (β = 10.71, SE = 4.1, *p*-value = 0.01).

**Table 2:** Linear regression of continuous birthweight by race, ethnicity and sexual orientation, National Survey of Family Growth (NSFG), 2006–2019 (*N* = 28,504)

	Unadjusted			Adjusted^a^		
	*B*	SE	P	*B*	SE	*P*
Total Population						
Heterosexual-WSM	0.00	(Reference)		0.00	(Reference)	
Heterosexual-WSW	-0.51	0.8	0.550	-0.11	0.8	0.890
Bisexual	-0.85	1.0	0.420	0.48	1.0	0.640
Lesbian	1.51	2.6	0.570	2.98	2.5	0.240
Non-Hispanic White						
Heterosexual-WSM	0.00	(Reference)		0.00	(Reference)	
Heterosexual-WSW	0.09	1.0	0.930	0.75	0.9	0.450
Bisexual	-1.57	1.4	0.290	0.17	1.4	0.910
Lesbian	-2.13	5.6	0.700	0.07	5.6	0.990
Non-Hispanic Black						
Heterosexual-WSM	0.00	(Reference)		0.00	(Reference)	
Heterosexual-WSW	-3.23	2.0	0.110	-3.16	2.0	0.130
Bisexual	1.02	1.7	0.570	1.54	1.7	0.380
Lesbian	2.61	3.5	0.470	3.05	3.5	0.390
Hispanic						
Heterosexual-WSM	0.00	(Reference)		0.00	(Reference)	
Heterosexual-WSW	-3.46	1.9	0.080	-2.61	1.9	0.170
Bisexual	-0.13	1.8	0.940	0.42	1.9	0.830
Lesbian	11.35	3.5	0.001	10.71	4.1	0.010

^a^Adjusted for maternal age, education, federal poverty level, nativity, previous preterm birth

In unstratified adjusted models (Table 3), pregnancies to heterosexual-WSW were significantly less likely to be preterm (aRR = 0.77, 95% CI 0.64,0.92) than those of heterosexual-WSM. There were no significant differences in babies born with low birthweight by sexual minority status in the full sample. However, bisexual women were significantly more likely to have a pregnancy resulting in miscarriage (aRR = 1.34, 95% CI 1.16,1.54) than pregnancies of heterosexual-WSM. Pregnancies to bisexual women were not significantly more likely to end in stillbirth after adjustment (aRR = 1.27, 95% CI 0.86,1.87). Pregnancies to lesbian women were more likely to end in stillbirth (aRR = 3.58, 95% CI 1.30,9.79) however, compared to heterosexual-WSM.

**Table 3:** Logistic regression of birth outcomes by sexual orientation, National Survey of Family Growth (NSFG), 2006–2019. N = 49,063 – 37,538)

	Total Population			
	Unadjusted		Adjusted^a^	
	RR	95% CI	RR	95% CI
Preterm birth (*N* = 37,538)				
Heterosexual-WSM	1.00	(Reference)	1.00	(Reference)
Heterosexual-WSW	0.77	(0.62, 0.94)	0.77	(0.64, 0.92)
Bisexual	0.91	(0.72, 1.15)	1.02	(0.83, 1.24)
Lesbian	0.58	(0.33, 1.02)	0.87	(0.53, 1.42)
Low birthweight (*N* = 35,229)				
Heterosexual-WSM	1.00	(Reference)	1.00	(Reference)
Heterosexual-WSW	1.01	(0.80, 1.27)	0.99	(0.79, 1.24)
Bisexual	1.33	(1.00, 1.78)	1.2	(0.89, 1.61)
Lesbian	1.03	(0.59, 1.80)	0.95	(0.56, 1.62)
Miscarriage (*N* = 49,063)				
Heterosexual-WSM	1.00	(Reference)	1.00	(Reference)
Heterosexual-WSW	1.18	(0.97, 1.42)	1.12	(0.93, 1.35)
Bisexual	1.34	(1.17, 1.55)	1.34	(1.16, 1.54)
Lesbian	1.9	(0.87, 4.19)	1.95	(0.94, 4.08)
Stillbirth (*N* = 38,549)				
Heterosexual-WSM	1.00	(Reference)	1.00	(Reference)
Heterosexual-WSW	1.22	(0.84, 1.75)	1.12	(0.77, 1.63)
Bisexual	1.5	(1.03, 2.19)	1.27	(0.86, 1.87)
Lesbian	4.09	(1.41, 11.89)	3.58	(1.30, 9.79)

*RR* risk ratio

^a^Adjusted for maternal age, education, Federal poverty level, nativity, previous preterm birth

Lastly, in Table 4, we found that pregnancies for NH White heterosexual-WSW were significantly less likely to end in preterm birth compared to heterosexual-WSM (aRR = 0.71, 95% CI 0.56,0.90). The increased risk of pregnancy ending in a low birthweight baby for bisexual NH White women (aRR = 1.44, 95% CI 0.95, 2.19) was not statistically significant after adjustment. Pregnancies to bisexual NH White women had a higher risk of ending in miscarriage (aRR = 1.31, 95% CI 1.11,1.55) than heterosexual-WSM. Lesbian NH White women also had a higher risk of their pregnancy ending in miscarriage (aRR = 2.39, 95% CI 1.31,4.38) stillbirth (aRR = 5.89, 95% CI 1.64,21.20) than heterosexual-WSM. No significant differences were noted in risk of adverse birth outcomes by sexual orientation for NH Black or Hispanic women.

**Table 4:** Logistic regression of birth outcomes by sexual orientation, race and ethnicity, NSFG 2006–2019 *N* = 49,063 – 37,538)

	Non-Hispanic White				Non-Hispanic Black				Hispanic			
	Unadjusted		Adjusted^a^		Unadjusted		Adjusted^a^		Unadjusted		Adjusted^a^	
	RR	95% CI	RR	95% CI	RR	95% CI	RR	95% CI	RR	95% CI	RR	95% CI
Preterm birth												
Heterosexual-WSM	1.00	(Reference)	1.00	(Reference)	1.00	(Reference)	1.00	(Reference)	1.00	(Reference)	1.00	(Reference)
Heterosexual-WSW	0.71	(0.55, 0.94)	0.71	(0.56, 0.90)	0.85	(0.57, 1.29)	0.93	(0.65, 1.33)	0.70	(0.40, 1.23)	0.63	(0.35, 1.15)
Bisexual	0.94	(0.70, 1.26)	0.97	((0.75, 1.24)	0.81	(0.50, 1.30)	1.01	(0.66, 1.53)	0.83	(0.44, 1.57)	1.16	(0.62, 2.17)
Lesbian	0.48	(0.21, 1.11)	0.74	(0(0.32, 1.69)	0.70	(0.28, 1.61)	0.93	(0.49, 1.75)	0.52	(0.13, 2.07)	0.83	(0.22, 3.61)
Low birthweight												
Heterosexual- WSM	1.00	(Reference)	1.00	(Reference)	1.00	(Reference)	1.00	(Reference)	1.00	(Reference)	1.00	(Reference)
Heterosexual-WSW	1.06	(0.77, 1.45)	0.99	(0.74, 1.35)	1.09	(0.74, 1.62)	1.14	(0.77, 1.65)	0.72	(0.42, 1.23)	0.75	(0.45, 1.27)
Bisexual	1.67	(1.11, 2.53)	1.1.44	0.95, 2.19)	0.94	(0.65, 1.35)	0.91	(0.63, 1.31)	0.83	(0.47, 1.47)	0.84	(0.47, 1.50)
Lesbian	1.15	(0.42, 3.02)	1.02	(0(0.40, 2.59)	1.04	(0.56, 1.94)	0.97	(0.52, 1.82)	0.13	(0.02, 1.05)	0.14	(0.02, 1.04)
Miscarriage												
Heterosexual- WSM	1.00	(Reference)	1.00	(Reference)	1.00	(Reference)	1.00	(Reference)	1.00	(Reference)	1.00	(Reference)
Heterosexual-WSW	1.12	(0.89, 1.42)	1.12	(0(0.89, 1.41)	1.20	(0.80, 1.80)	1.16	(0.79, 1.71)	1.12	(0.85, 1.48)	0.97	(0.74, 1.26)
Bisexual	1.29	(1.10, 1.53)	1.31	(1.11, 1.55)	1.27	(0.87, 1.85)	1.33	(0.92, 1.93)	1.32	(0.93, 1.86)	1.18	(0.84, 1.66)
Lesbian	2.41	(1.25, 4.62)	2.39	(1.31, 4.38)	0.76	(0.35, 1.63)	0.76	(0.35, 1.66)	0.47	(0.18, 1.24)	0.55	(0.21, 1.45)
Stillbirth												
Heterosexual- WSM	1.00	(Reference)	1.00	(Reference)	1.00	(Reference)	1.00	(Reference)	1.00	(Reference)	1.00	(Reference)
Heterosexual-WSW	1.32	(0.82, 2.14)	1.19	(0.73, 1.93)	1.11	(0.58, 2.13)	1.17	(0.60, 2.26)	1.14	(0.38, 3.47)	0.91	(0.27, 3.03)
Bisexual	1.46	(0.88, 2.42)	1.17	(0.70, 1.97)	1.57	(0.84, 2.94)	1.54	(0.81, 2.93)	1.22	(0.48, 3.08)	1.00	(0.37, 2.67)
Lesbian	7.21	(1.65, 31.53)	5.89	(1.64, 21.20)	1.33	(0.52, 3.38)	1.31	(0.51, 3.34)	3.10	(0.91, 10.5)	3.04	(0.91, 10.1)

*RR* risk ratio

^a^Adjusted for maternal age, education, Federal poverty level, nativity, previous preterm birth

## Discussion

In a nationally representative sample, we found variation in the risk of adverse pregnancy outcomes by sexual orientation, race, and ethnicity. Evidence suggests that sexual minority women are at greater risk of adverse pregnancy outcomes [10], and we tested the hypothesis that multiple marginalized identities would confer even greater risk. Though we did not find consistent support of our hypothesis, we instead report a more complex relationship, indicating the importance of future work using an intersectional approach that also queries the structures and system underlying social and health inequities.

We found that babies born to lesbian Hispanic women had significantly higher birthweights than heterosexual-WSM. This is distinct from the previous finding that lesbians, not stratified by race, had significantly greater odds of low birthweight (OR 2.64, 95% CI 1.38–5.07) [10], suggesting the identification of an important point of within-group variation and the need for further research to examine the distribution of birthweights among different populations. In the general population, Hispanic women have rates of singleton low birthweight similar to white women (6.04% vs 5.21%) [3]. These findings differ from those recently published with the National Longitudinal Study of Adolescent to Adult Health (Add Health), which found that SMW of color (both NH Black and Hispanic) had an increased risk of preterm birth and low birthweight [24]. There are several possible reasons for this divergence; first, the Add Health study was prospective and allows for correct time ordering of sexual identity prior to a pregnancy. NSFG data measures sexual orientation at the time of survey, which may not correspond with a respondent's identity at the time of birth. Exposure to stigma and its implications for birth outcomes may vary across these samples. Relatedly, the prospective nature of the Add Health data means that the study could adjust for health behaviors and socioeconomic status prior to the pregnancy. Finally, though NSFG is a much larger sample of women than Add Health, the sample sizes of Hispanic SMW are relatively small, and may indicate that our analyses reflect sampling bias which may have driven some of the results. Given the paucity of research in this area, more studies are needed across multiple data sets to reconcile differences across these samples. Further, national studies of pregnancy and birth should all include measures of sexual and gender minority status, and we recognize efforts to achieve the important goal and to address the significant gap in data quality and availability for this understudied and vulnerable population.

In this sample, bisexual women had a higher risk of miscarriage than their heterosexual-WSM counterparts. This may be explained by the large proportion of this sample that was NH White driving the findings, as NH Black and Hispanic women had a lower risk of miscarriage, though these effects were not statistically significant. NH White lesbian women also had a higher risk of miscarriage that NH White heterosexual-WSM, though again this was not observed among NH Black or Hispanic women. Both the limited sample size of Hispanic and NH Black SMW in this study sample and the inherent challenges in identifying and measuring miscarriage may influence these findings. Women who do not realize they are pregnant may think they are having a regular or somewhat mistimed menstrual period, and not recognize that a miscarriage has occurred. It may be relevant that early pregnancy recognition is typical with assistive reproductive technology (ART), and a significantly greater portion of lesbians used ART than heterosexual women who had sex with men in our sample (3.12% vs 1.79%) [26]. However, the influence on reported miscarriage rates is multi-directional: the expense of ART reduces may lower the use of this technology NH Black women with lower socioeconomic status, reducing early recognition, but ART procedures themselves are associated with racially disparate increased rates of adverse outcomes [27]. The complex interplay between intersectional minority stress, access to ART, risk of miscarriage associated with ART procedures, and the limitations of miscarriage surveillance warrant further investigation.

We observed higher rates of stillbirth overall for lesbians compared to heterosexual-WSM, though again, these findings were not observed in stratified analyses among NH Black and Hispanic women. Risk of stillbirth was attenuated in the multivariable model, where use of ART was a significant driver, yet still significant. One potential explanation for the high rates is the self-reported nature of the miscarriage, abortion and stillbirth data [28] and the lay public’s lack of understanding of the nuances of measuring these outcomes. The addition of sexual orientation to population-based surveys and surveillance mechanisms such as the Pregnancy Risk Assessment Monitoring System (PRAMS) would permit replication of these findings, free from any error or bias introduced by self-report. Further, this work highlights the urgent need for additional research using larger samples of NH Black and Hispanic SMW women, careful report of ART use, and other institutional and structural factors that may influence outcomes.

This study had several strengths and limitations. As a nationally representative study, the NSFG sampling methods can provide reasonable assurance that findings reflect actual rates in the population, especially for NH White SMW, who are well-represented. Another strength is that this study is one of the few national studies with information on sexual orientation and multiple indicators of pregnancy outcomes. A potential limitation is that birth outcomes are self-reported, although women have been shown to be reliable in reporting these outcomes in previous large studies [29]. This study is also limited by a lack of measures that could be used to assess minority stress, racism and discrimination, and number of prenatal care visits. While some covariates can be assumed to be temporally stable and identical for all pregnancies (i.e., racial and ethnic identity), it is a limitation that mutable characteristics were collected at the time of study interviews, not at the time of pregnancy. It would be particularly valuable to know socioeconomic markers, including educational attainment and poverty status, at the time of pregnancy. We also did not have data on current smoking status, which may result in residual confounding. Further, the single binary measure of ART collapses a wide array of medical procedures that can vary in their contribution of risk towards adverse obstetrical outcomes. Future research should be prospectively collected and carefully measure history of ART use and outcomes. There is a possibility of selection bias, as those who participate in such a study may have better outcomes than those in the general population. These data do not assess participants’ gender identity, which may interact with sexual orientation and racial and ethnic background to influence adverse birth outcomes.

## Conclusions

In this sample, pregnancies in heterosexual-WSW were less likely to end in preterm birth than in heterosexual-WSM. Pregnancies to lesbians and bisexual women were more likely to end in miscarriage or stillbirth than heterosexual WSM, however, these findings appear to be driven primarily by NH White women in this sample. NH Black and NH Hispanic LGB women did not consistently have worse outcomes than heterosexual White women, in fact, lesbian Hispanic women had babies with higher birthweights compared to heterosexual-WSM Hispanic women. Future research on pregnancy and birth outcomes in SMW should expand their focus beyond individual-level behaviors and risks to include institutional and structural factors. This approach may offer a more comprehensive examination of the problem and opportunities for intervention.

## Data Availability

The datasets analyzed during the current study are publicly available from the National Survey of Family Growth, https://www.cdc.gov/nchs/nsfg/nsfg_2017_2019_puf.htm

## Abbreviations

US: United States
NH: Non-Hispanic
SMW: Sexual Minority Women
LGBT: Lesbian, Gay, Bisexual, Transgender
NSFG: National Survey of Family Growth
CAPI: Computer Assisted Personal Interview
FPL: Federal Poverty Line
WSM: Women who have sex with men
WSW: Women who have sex with women
PRAMS: Pregnancy Risk Assessment Monitoring System
RR: Relative risk
aRR: Adjusted relative risk
CI: Confidence interval
Β: Beta value
SE: Standard error
OR: Odds ratio
ART: Assisted reproductive technology
